# Biodegradation and Biotransformation of Indole: Advances and Perspectives

**DOI:** 10.3389/fmicb.2018.02625

**Published:** 2018-11-01

**Authors:** Qiao Ma, Xuwang Zhang, Yuanyuan Qu

**Affiliations:** ^1^Institute of Environmental Systems Biology, College of Environmental Science and Engineering, Dalian Maritime University, Dalian, China; ^2^Key Laboratory of Industrial Ecology and Environmental Engineering (Ministry of Education), School of Food and Environment, Dalian University of Technology, Panjin, China; ^3^Key Laboratory of Industrial Ecology and Environmental Engineering (Ministry of Education), School of Environmental Science and Technology, Dalian University of Technology, Dalian, China

**Keywords:** indole, signaling molecule, biodegradation, biotransformation, indigo, functional study

## Abstract

Indole is long regarded as a typical *N*-heterocyclic aromatic pollutant in industrial and agricultural wastewater, and recently it has been identified as a versatile signaling molecule with wide environmental distributions. An exponentially growing number of researches have been reported on indole due to its significant roles in bacterial physiology, pathogenesis, animal behavior and human diseases. From the viewpoint of both environmental bioremediation and biological studies, the researches on metabolism and fates of indole are important to realize environmental treatment and illuminate its biological function. Indole can be produced from tryptophan by tryptophanase in many bacterial species. Meanwhile, various bacterial strains have obtained the ability to transform and degrade indole. The characteristics and pathways for indole degradation have been investigated for a century, and the functional genes for indole aerobic degradation have also been uncovered recently. Interestingly, many oxygenases have proven to be able to oxidize indole to indigo, and this historic and motivating case for biological applications has attracted intensive attention for decades. Herein, the bacteria, enzymes and pathways for indole production, biodegradation and biotransformation are systematically summarized, and the future researches on indole-microbe interactions are also prospected.

## Introduction

Indole, which was firstly isolated from indigo reduction process, is a typical nitrogen heterocyclic aromatic compound widespread in our daily products and natural environment. Indole ring is present in many alkaloids, phytohormones, plant flower oils, pigments and proteins (de Sá Alves et al., 2009; Arora et al., 2015). Since indole nucleus has a wide spectrum of biological activities, including anticancer, antiviral, antimicrobial, anti-inflammatory, antiHIV and antidiabetic activities, it is extensively used in the pharmaceutical industries (Gribble, 2010; Sharma et al., 2010). Plants and microorganisms can produce indole by tryptophanase or its analogs in the presence of indole-3-glycerol phosphate and tryptophan, thus indole is present in plants and bacteria-rich niches, such as soil, rhizosphere, sludge and intestinal tracts (Frey et al., 2000; Lee and Lee, 2010). In addition, relatively high concentration of indole has been found in coal tar and animal feces and sewage, constituting the major contributor to odor pollution (Yamamoto et al., 1991; Mackie et al., 1998).

Indole has long been regarded as a typical *N*-heterocyclic aromatic pollutant due to its toxicity and potential mutagenicity. Studies have shown that indole can cause animal hemolysi, hemoglobinuric nephrosis, temporary skin irritation, tumor formation and plant low pigmentation (Collin and Höke, 2000; Arora et al., 2015). For microorganisms, indole can induce bacterial membrane and oxidant toxicity, prevent cell division by modulating membrane potential, inhibit adenosine triphosphate production and protein folding, and cause reparable DNA damage (Osawa et al., 1983; Garbe et al., 2000; Chimerel et al., 2012; Kim J. et al., 2013). Therefore, the biodegradation and bioremediation of indole have been concerned over a century.

Recently, indole has been proven to be a versatile interspecies and interkingdom signaling molecule which plays important roles in prokaryotic and eukaryotic communities (Lee et al., 2015a). Indole can affect the spore formation, plasmid stability, cell division, antibiotic tolerance, biofilm formation and virulence of both indole-producing and non-indole-producing microorganisms (Lee and Lee, 2010). Indole can be produced by plants (e.g., maize) upon herbivore attack, which will act as the aerial priming agent to warn their non-attacked tissues and neighboring plants for the imminent attack (Erb et al., 2015). Indole can also control the behavior of insects. More importantly, increasing evidences indicate that indole is rich in human intestinal tracts and closely related with immune systems and several human diseases (Lee et al., 2015a). Overall, the function study of indole is still in initial stage and much is awaiting to be discovered and investigated.

Since indole is widely distributed in different environment matrices, it will encounter virous kinds of microorganisms. Many bacteria have acquired the ability to transform and degrade indole to improve their survival state. The researches on biotransformation and biodegradation of indole are of importance to realize environmental bioremediation and illuminate its biological functions. Herein, this review presents the history and recent advances regarding indole biotransformation and biodegradation, and the research trends in indole-microbe studies are also prospected.

## Indole is widespread in nature

**Figure 1:**
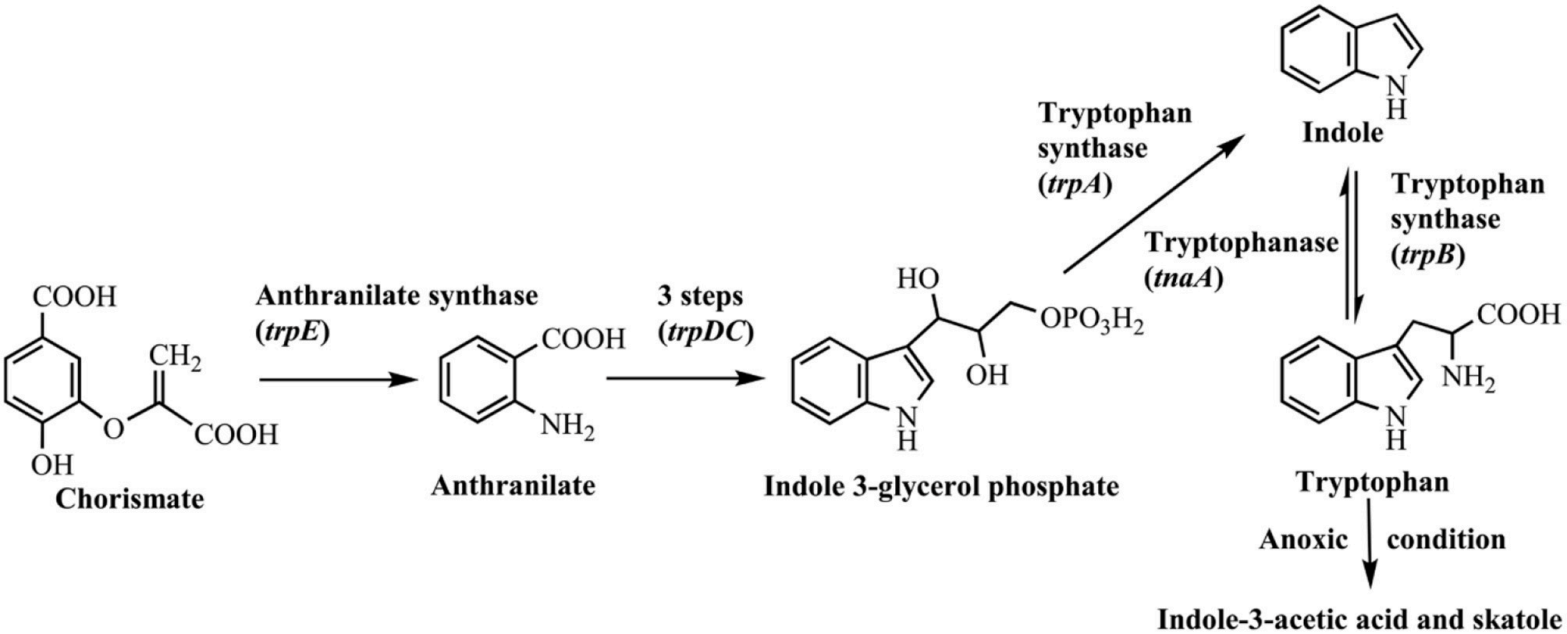
Indole synthesis pathway in bacteria.

Indole was firstly discovered in indigo reduction process by Baeyer in the year of 1866, which was then successfully synthesized by Baeyer (1866) and Fischer and Jourdan (1883). In 1897, it was found that bacterial strains, including *Escherichia coli* and *Vibrio cholerae*, could produce indole, and this feature has long been used for strain identification (Smith, 1897). The synthetic pathways for indole in bacteria are summarized in Figure 1. It is well known that biosynthesis of tryptophan is catalyzed by TrpABCDE encoded by the tryptophan operon. Tryptophan synthase is encoded by *trpA* and *trpB*, and catalyzes the hydrolysis of indole 3-glycerol phosphate to indole and the subsequent condensation of indole and *L*-serine to form *L*-tryptophan. The intermediate indole is transferred from the alpha-subunit to the beta-subunit and not released from the tryptophan synthase complex (Murdock et al., 1993). Another indole-related pathway is catalyzed by tryptophanase (TnaA), which can reversibly convert tryptophan to indole, pyruvate and ammonia (Watanabe and Snell, 1972). This is the sole indole-producing pathway in bacteria to date, and over 85 bacterial species (both Gram-negative and Gram-positive bacteria) can produce indole from tryptophan (Lee and Lee, 2010). The indole-producing bacteria occupy various environmental niches, suggesting indole can exist widespread such as in activated sludge, soil, plant rhizosphere, ocean, animals and livestock waste. It is noted that tryptophan can also be converted to indole-3-acetic acid and 3-methylindole (skatole), especially by gut bacteria under anoxic condition, whereas the mechanism and regulation of indole and skatole production remain unclear (Mackie et al., 1998; Deslandes et al., 2001).

Generally, the indole concentration can reach 0.5–0.6 mM when *E. coli* and *V. cholerae* are cultured in Luria-Bertani (LB) medium, and 0.25–1.1 mM in the mouse and human gut (Kobayashi et al., 2006; Mueller et al., 2009; Lee et al., 2015a). Several environmental and biological factors have been proven to affect the indole production level, of which the amount of exogenous tryptophan is the most important one. Li and Young (2013) found that the *E. coli* converted the tryptophan into an equal amount of indole up to 5 mM, and the synthesis process mainly depended on the tryptophan importer protein TnaB. However, further increase of tryptophan would not accumulate indole. The possible reason might be that excess indole inhibited TnaA enzyme activity and tryptophan transport process. High pH and temperature promoted indole production. Indole yield increased with pH value in the range of 4.0–9.0 since low pH inhibited TnaA expression, and TnaA was highly expressed at pH 9.0 (Han et al., 2011). Surprisingly, *E. coli* strain produced nearly 7 mM indole/cell OD at 50°C, 89-fold higher than that at 37°C, though it stopped growing at such high temperature. When *E. coli* strains exposed to antibiotics, such as ampicillin and kanamycin, the indole yield also significantly increased, which should be beneficial for the resistance and cell survival against antibiotics (Han et al., 2011).

**Figure 2:**
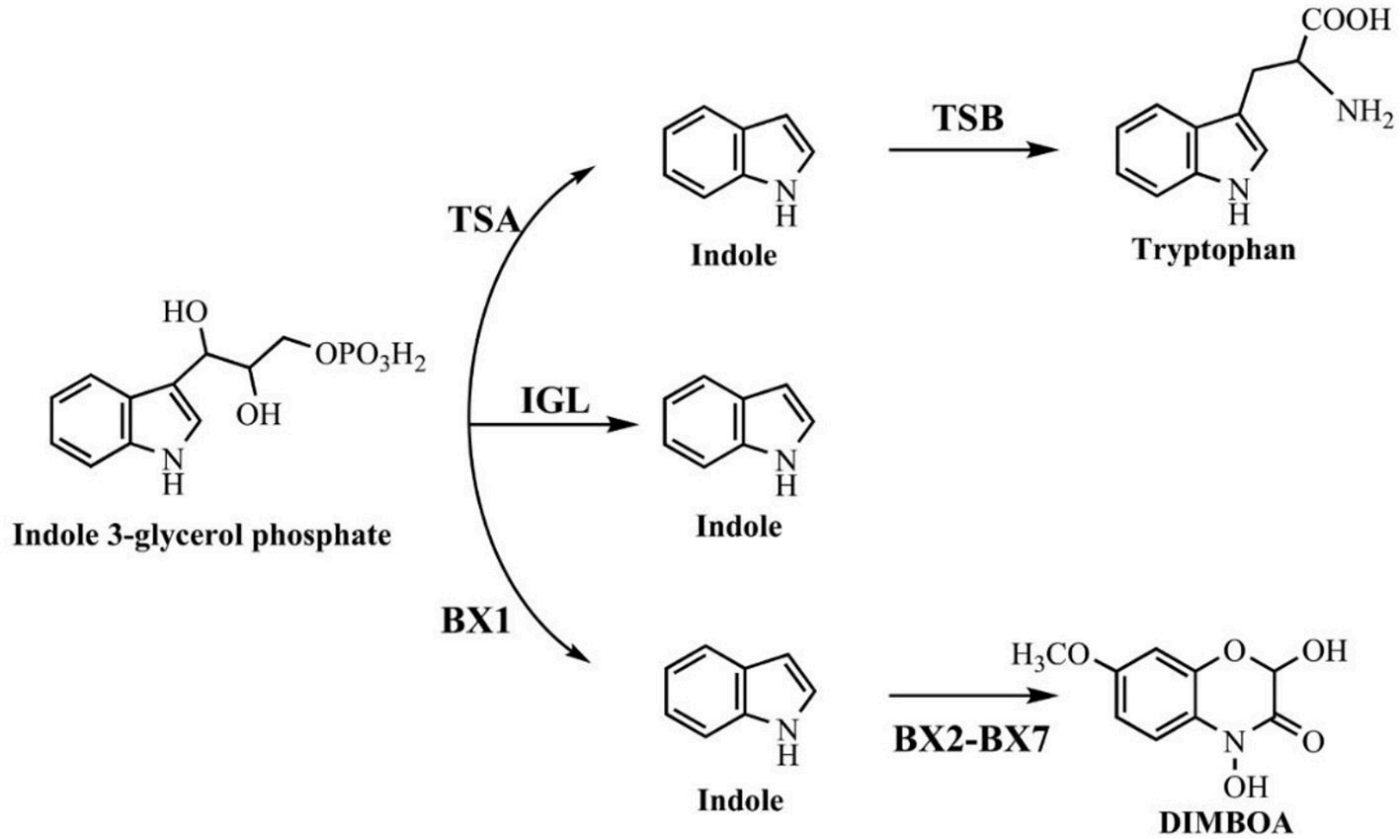
Indole synthesis pathway in plants.

Indole is also found in a variety of natural plant flower oils, such as jasmine and daffodils. It is reported that natural jasmine oils contain as high as 2.5% indole (Collin and Höke, 2000). Many plants, including cotton, corn, green beans, cucumbers and apple trees, can release volatile organic compounds to protect themselves from insect threats, and indole is one of the typical secondary metabolites (Erb et al., 2015). Three pathways relevant to indole production in plants have been reported as shown in Figure 2. The typical tryptophan biosynthetic route encoded by *trp* operon was also conserved in plants. Plant tryptophan synthase α (TSA) and tryptophan synthase β (TSB) showed high similarities with those in bacteria and catalyzed the conversion of indole-3-glycerol phosphate to tryptophan without indole release (Radwanski and Last, 1995). The enzymes encoded by gene cluster *bx* can catalyze the production of cyclic hydroxamic acid 2,4-dihydroxy-7-methoxy-2H-1,4-benzoxazin-3(4H)-one (DIMBOA) from IGP in plants. Frey et al. (1997) demonstrated that the first enzyme BX1 in the cluster was a TSA homolog, and could directly catalyze the indole production from IGP independent of any TSB. Then another indole-producing enzyme, i.e., indole-3-glycerol phosphate lyase (IGL), was identified in maize (Frey et al., 2000). Sequence analysis indicated that both IGL and BX1 should be derived from TSA.

## Indole is produced by anthropogenic activities

Anthropogenic activities significantly promote the distribution of indole in the environment. Indole is chemically synthesized in technical quantities as a feedstock or precursor in the production of tryptophan, plant growth regulators, dyes and pharmaceuticals (Barden, 2010). Indole has been found in coal tar over 100 years (Collin and Höke, 2000), and with the development of coal mining, petroleum processing and tobacco industries, plenty of indole-containing wastewater and smoke is produced and discharged into the environment. On the other hand, the increasing urbanization and intensification of animal production throughout the world have resulted in serious animal waste and odor nuisance. Indole and skatole produced by intestinal anaerobes from tryptophan have been implicated as a major factor in odor nuisance and livestock pollution, and they are released in the environment through gas transmission, livestock wastewater discharge and biosolids application to agricultural lands (Mackie et al., 1998; Kinney et al., 2006).

## Indole degradation by aerobic bacteria

### Indole aerobic degradation bacteria and pathways

Research on indole degradation initiated from the 1920s and the reported indole-degrading strains are summarized in Table 1. Raistrick and Clark (1921) firstly proved that *Bacillus pyocyaneus, B. fluorescens* and *B. prodigiosus* could attack indole nucleus and release ammonia, and Supniewski (1923) further stated that *B. pyocyaneus* could degrade indole in a low rate. Gray (1928) obtained the *Pseudomonas indoloxidans* strain from soil which produced indigo in the process of indole decomposition, but it could not utilize indole as the sole carbon source. Detailed analysis of indole aerobic degradation was started by Sakamoto et al. (1953), who firstly proposed a reasonable indole degradation pathway as indole-indoxyl-isatin-*N*-formylanthranilate-anthranilate-salicylate-catechol by a Gram-negative bacterium. Fujioka and Wada (1968) then isolated a Gram-positive bacterium and obtained a dihydroxyindole oxygenase which directly catalyzed dihydroxyindole to anthranilic acid. Results also showed that the strain could not decompose *N*-formylanthranilate, isatin and 2-oxindole, indicating it might convert dihydroxyindole to anthranilate via a single enzyme step. However, the enzyme catalyzing the first step of indole oxidation was not obtained, which was associated with cellular debris after high-speed centrifugation. In Claus and Kutzner (1983), isolated an *Alcaligenes* spec. In3 and proposed the indole-indoxyl-isatin-anthranilate-gentisate pathway, while the gentisate formation process from anthranilate remained to be elucidated. In 1997, *P*. sp. ST-200 was reported to be able to assimilate high concentration of indole dissolved in organic solvent, and isatin and isatic acid were detected as the main intermediates (Doukyu and Aono, 1997). Strain ST-200 could not utilize isatic acid as the growth substrate, suggesting it assimilated indole in another degradation pathway. Another pseudomonad strain GS was isolated from mangrove sediment and produced two novel metabolites without further identification (Yin et al., 2005).

**Table 1:** Summary of indole-degrading bacteria.

**Bacteria**	**Features**	**References**
*A. baumannii* ATCC19606	Identification of an *iif* gene cluster for the first time	Lin et al., 2015
*A*. sp. O153	Identification of the *iif* gene cluster and indole degradation pathway	Sadauskas et al., 2017
*A. pittii* L1	Production of 4-(3-hydroxy-1H-pyrrol-2-yl)-2-oxo-but-3-enoic acid and isatin as the intermediates	Yang et al., 2017
*Agrobacterium tumefaciens*	Degradation of indole rapidly in LB medium	Lee et al., 2015b
*Alcaligenes* spec. In3	Identification of the indole-indoxyl-isatin-anthranilate-gentisate pathway	Claus and Kutzner, 1983
*Alcaligenes faecalis* IITR89	Production of indigo in indole degradation and the genome is sequenced	Regar et al., 2016
*Alcaligenes* sp. B5	Degradation of 1.0 mM indole in 16 h and produce anthranilic acid and isatin	Kim et al., 2016
*Arthrobacter* sp. B1	Production of anthranilic acid and isatin	Kim et al., 2016
*Arthrobacter* sp. SPG	Production of indole-3-acetic acid, indole-3-glyoxylic acid, and indole-3-aldehyde	Arora and Bae, 2014
*B*. spp.	First report on indole degradation	Raistrick and Clark, 1921
*Burkholderia unamae* CK43B	Degradation of indole in the presence of pyrogallol-type polyphenols	Kim D. et al., 2013
*C*. sp. KK10	First report of indole carbocyclic-aromatic ring cleavage pathway	Fukuoka et al., 2015
*C*. sp. SHE	Identification of *ind* gene cluster and indole degradation pathway	Qu et al., 2015
*C*. sp. IDO	Degradation of indole efficiently and the genome is sequenced	Ma et al., 2015a
Unidentified gram-negative bacterium	Identification of the indole-indoxyl-isatin-N-formylanthranilate-anthranilate-salicylate-catechol pathway	Sakamoto et al., 1953
Unidentified gram-positive bacterium	Purification of a dihydroxyindole oxygenase for anthranilic acid formation	Fujioka and Wada, 1968
*P. aeruginosa*	Degradation of indole rapidly in LB medium	Lee et al., 2009
*P. indoloxidans*	Production of indigo in indole decomposition process	Gray, 1928
*P*. sp. Gs	Production of two novel metabolites	Yin et al., 2005
*P*. sp. ST-200	Assimilation of indole in water-organic solvent two phase system, and isatin and isatic acid are generated	Doukyu and Aono, 1997
*Thauera* sp. Q4/Q20-C/3–35	Degradation of indole, phenol, and methylphenol under aerobic conditions	Mao et al., 2010
*Desulfobacterium indolicum* In04	First reported anaerobic indole-degrading strain	Bak and Widdel, 1986
*P*. sp. LPA11/LPB11/LPC24	Degradation of indole via oxindole-isatin-anthranilic acid pathway under anoxic condition	Li et al., 2009

As the biological functions of indole have been extensively reported in the last decade, indole biotransformation studies have received more attention. Recently, Fukuoka et al. (2015) characterized the indole biotransformation pathway in *Cupriavidus* sp. KK10. It could degrade indole not only via *N-*heterocyclic ring cleavage pathway but also carbocyclic-aromatic ring cleavage pathway with initial attack at C4 and C5 positions. We have recently obtained two *Cupriavidus* strains named IDO and SHE from indole acclimated samples. They could utilize indole as sole carbon source and efficiently remove 100 mg/L indole within a day when cultured in simple mineral salt medium at 30°C and 150 r/min (Ma et al., 2015a; Qu et al., 2015). Intermediate analyses indicated that strain SHE produced isatin, isatic acid, and anthranilate in indole degradation process (Qu et al., 2017). As for *Acinetobacter*, Lin et al. (2015) demonstrated that strain *A. baumannii* ATCC19606 could detoxify indole by converting it to non-toxic indigo, and the functional genes responsible for indole oxidation were firstly identified and characterized. Subsequently, Sadauskas et al. (2017) reported that strain *A*. sp. O153 degraded indole via the indole-2,3-dihydrodiol, 3-hydroxyindolin-2-one, and anthranilate step through a similar *iif* gene cluster with that in strain ATCC19606. Yang et al. (2017) isolated an *A. pittii* L1 from coking wastewater and identified the 4-(3-hydroxy-1H-pyrrol-2-yl)-2-oxo-but-3-enoic acid and isatin as the intermediates, suggesting that strain L1 could attack both the carbocyclic and *N-*heterocyclic rings, similar with that in strain *C*. sp. KK10. In addition to *Cupriavidus* and *Acinetobacter*, other recently reported indole-degrading strains included *Arthrobacter* sp., *Alcaligenes* sp., *Burkholderia unamae, P. aeruginosa*, and *Agrobacterium tumefaciens* (Lee et al., 2009, 2015b; Kim D. et al., 2013; Arora et al., 2015). It is noted that some clinically important bacterial species (e.g., *Providencia*) can produce and degrade indole simultaneously, and the metabolic process and biological significance of this phenomenon need further investigation (Müller, 1986).

**Figure 3:**
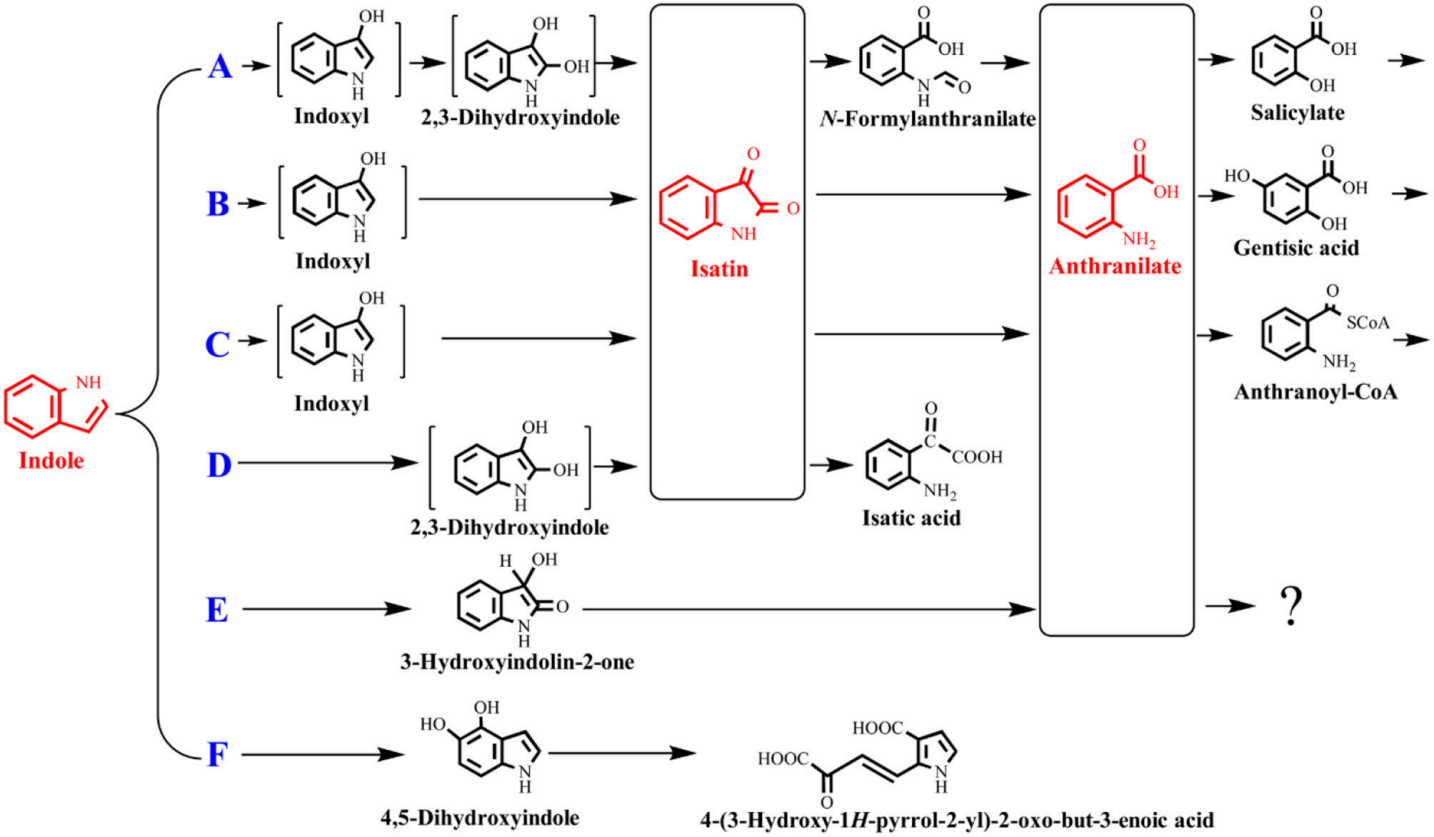
Summary of indole aerobic degradation pathways. **A**, a Gram-negative bacterium isolated from tap water (Sakamoto et al., 1953); **B**, *Alcaligenes* spec strain In 3 (Claus and Kutzner, 1983); **C**, *C*. sp. SHE (Qu et al., 2017); **D**, *P*. sp. ST-200 (Doukyu and Aono, 1997); **E**, a Gram-positive coccus (Fujioka and Wada, 1968) and *A*. sp. O153 (Sadauskas et al., 2017); **F**, *C*. sp. KK10 and *A. pittii* L1 (Fukuoka et al., 2015; Yang et al., 2017).

The indole degradation pathways are summarized in Figure 3, and it can be seen that isatin and anthranilate are the key intermediates in several studies (Qu et al., 2017). Isatic acid, the hydrolase product of isatin, was detected in *P*. sp. ST-200 and *C*. sp. SHE (Doukyu and Aono, 1997; Qu et al., 2015), further suggesting that isatin participated in indole degradation in these strains. However, studies in *A*. sp. O153 and a Gram-positive coccus suggested that anthranilate was directly formed from dihydroxyindole or 3-hydroxyindolin-2-one bypass isatin (E pathway in Figure 3; Fujioka and Wada, 1968; Sadauskas et al., 2017). Therefore, the roles of isatin in indole degradation should be further confirmed. Indole degradation via the carbocyclic ring cleavage pathway has been recently identified in *C*. sp. KK10 and *A. pittii* L1 (F pathway in Figure 3; Fukuoka et al., 2015; Yang et al., 2017), and the frequency of this pathway is worthy of further investigation. Moreover, the biotransformation of anthranilate, the key intermediate linking indole upstream and downstream metabolism, is diverse in different strains, and the understanding of anthranilate metabolic pathway is crucial to fully clarify the fate of indole in the microbial communities (Qu et al., 2017; Sadauskas et al., 2017).

### Functional genes for indole conversion

Identification of indole degradation related genes and gene clusters would undoubtedly enhance our understanding of its transformation process. Moreover, it can be inferred that indole oxygenase which utilized indole as the primary substrate may be advantageous over other aromatic oxygenases in indole biotransformation to form indigo. However, the functional genes responsible for indole degradation have remained a black box for a century. Many oxidoreductases, such as naphthalene dioxygenase, biphenyl dioxygenase, phenol hydroxylase and cytochrome P450 hydroxylase, could oxidize indole to indoxyl, which could be further dimerized into indigoids, while no evidences were shown that indole was the inherent substrate for these enzymes (Ensley et al., 1983; O'Connor et al., 1997; Kim et al., 2005). Fujioka and Wada (1968) have tried to purify the indole oxygenase, the key enzyme that catalyzed the first step reaction of indole. The target protein was not solubilized and the catalytic activity could not be determined since bacterial strain or cell debris oxidized indole to anthranilate directly. A dihydroxyindole oxygenase was unexpectedly obtained and it was proven to catalyze dihydroxyindole to anthranilate without formation of other intermediates, which should be the adjacent enzyme of indole oxygenase. Similarly, Kamath and Vaidyanathan (1990) tried to determine the indole oxygenase activity in *Aspergillus niger* under a series of conditions but ended in failure.

**Figure 4:**
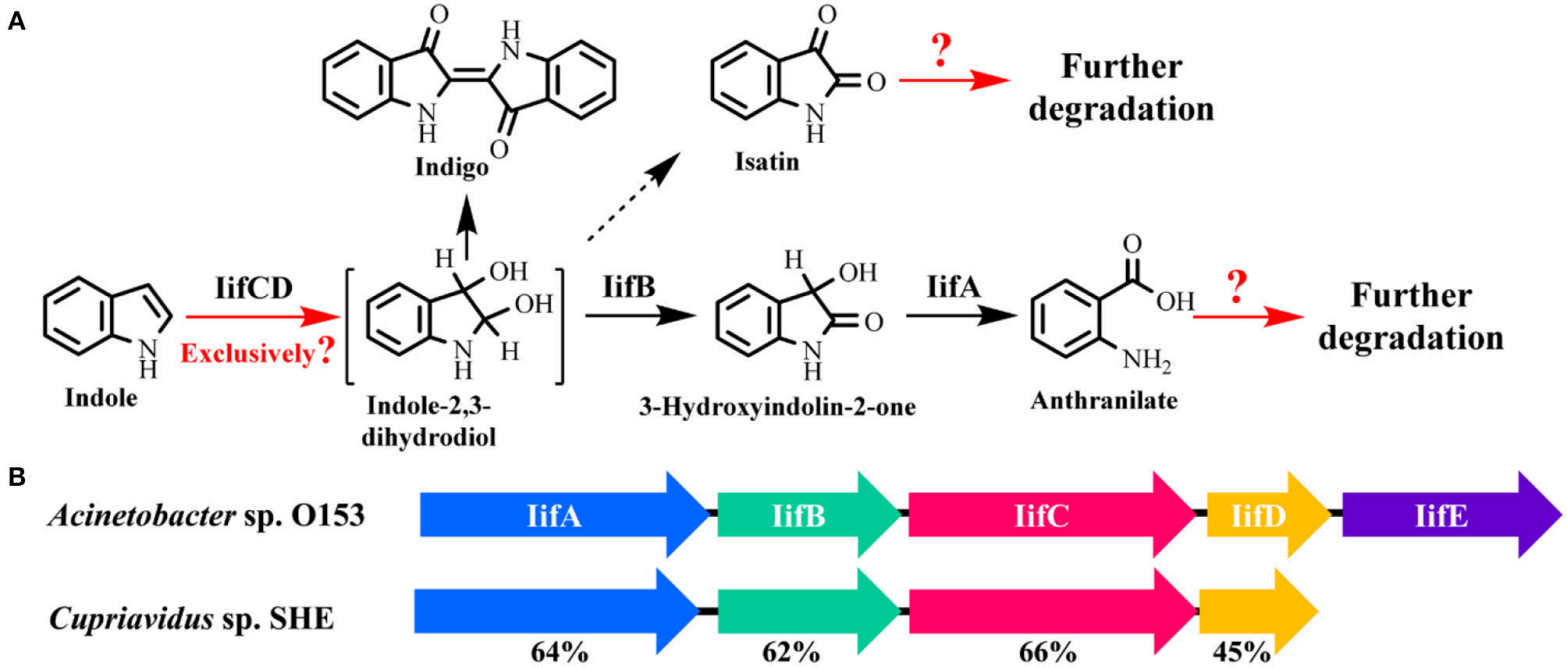
Summary of indole aerobic degradation functional enzymes. **(A)** overall indole degradation pathway and corresponding enzymes; **(B)** *iif* gene cluster organization in strain *A*. sp. O153 and *C*. sp. SHE.

Very recently, Sadauskas et al. (2017) and Qu et al. (2017) simultaneously reported the genetic determinants for indole degradation in *Acinetobacter* and *Cupriavidus*, respectively (Figure 4). They both identified an *iif* (or *ind*) gene cluster, which was widespread in a range of bacteria, responsible for indole upstream metabolism to produce anthranilate. The oxygenase and flavin reductase units encoded by *iifC* and *iifD*, which were firstly identified and characterized by Lin et al. (2015), constituted the two-component indole oxygenase system responsible for indole oxidation to *cis*-2,3-dihydrodiol. The short-chain dehydrogenase IifB converted indole-2,3-dihydrodiol to 3-hydroxyindolin-2-one. And then a cofactor-independent oxygenase IifA was proven to produce anthranilate directly from 3-hydroxyindolin-2-one. The role of *iifE* in the cluster was ruled out due to its unchanged expression level and unclear function in indole transformation process.

Several key questions still remain in this research. (i) Is *iif* the exclusive gene cluster for indole degradation? Answer should be no. One typical example is that the type strain *P. aeruginosa* PAO1 can degrade indole effectively, while genomic analysis indicates that this strain lacks *iif* related genes (Lee et al., 2009). In the *iif*-dependent degradation bacteria, more than one copy of *iif* gene cluster exists in some strains, such as *C*. sp. IDO (Ma et al., 2015a), thus the role of these clusters should be resolved. (ii) Is isatin involved in indole degradation? It was shown that there was no gene in the *iif* cluster associated with isatin production or transformation, thus Sadauskas et al. (2017) suggested that isatin should be a dead-end product or even an experimental artifact. Nevertheless, isatic acid was detected in some studies, indicating that the isatin was formed and further metabolized possibly by an isatin hydrolase. Further BLAST analysis indicates that the isatin hydrolase gene is spread in numerous strains, including indole-degrading genera *Cupriavidus, Burkholderia* and *Azoarcus*, and functional identification of this gene is worth investigation (Bjerregaard-Andersen et al., 2014). (iii) What is the functional gene cluster for anthranilate metabolism in indole degradation process? Anthranilate can be further degraded by four pathways, i.e., catechol pathway catalyzed by anthranilate-1,2-dioxygenase, gentisate pathway catalyzed by an unreported enzyme, 3-hydroxyanthranilate pathway catalyzed by anthranilate hydroxylase, and 2-aminobenzoyl-CoA pathway catalyzed by 2-aminobenzoayl-CoA ligase and 2-aminobenzoyl-CoA monooxygenase/reductase (Langkau et al., 1995; Arora, 2015). Sadauskas et al. (2017) and Lee et al. (2009) speculated that anthranilate might be degraded by the anthranilate dioxygenase by genomic and transcriptomic analyses. However, we proved that a 2-aminobenzoayl-CoA gene cluster was vital for anthranilate degradation by proteomic and genetic verification (Qu et al., 2017). Moreover, gentisate was detected as the intermediate in indole degradation (Claus and Kutzner, 1983), and the functional gene for gentisate pathway has not yet been revealed to date. (iv) How is the *iif* gene cluster regulated? Previous study indicated that an IifR protein was required for indole oxygenase expression in strain *A. baumannii* ATCC19606 (Lin et al., 2015). However, IifR was constitutively expressed irrespective of indole concentrations and the regulation mechanism was not further clarified.

### Mixed culture approach on indole degradation

Apart from pure culture studies, indole degradation by aerobic microbial communities was rarely investigated. In this respect, we have systematically explored microbial community composition and structure using flask and sequencing batch reactor systems by Illumina high throughput sequencing technique (Ma et al., 2015b; Zhang et al., 2015). *Azorcus* and *Thauera* were the dominant genera in the microbial community with indole and glucose as the carbon source. When indole was utilized as sole carbon and/or nitrogen source, *Alcaligenes, Burkholderia*, and *Comamonas* were the main compositions. Several strains from these genera have been reported previously, which should be valuable microbial resources for indole remediation. However, it was noted that indole degradation intermediates could be the carbon sources for many strains, thus the functional populations toward indole should be further affirmed. Besides, the functional genes in the microbial community for indole transformation have not been concerned.

## Indole degradation under anaerobic conditions

### Indole anaerobic degradation under various conditions

**Figure 5:**
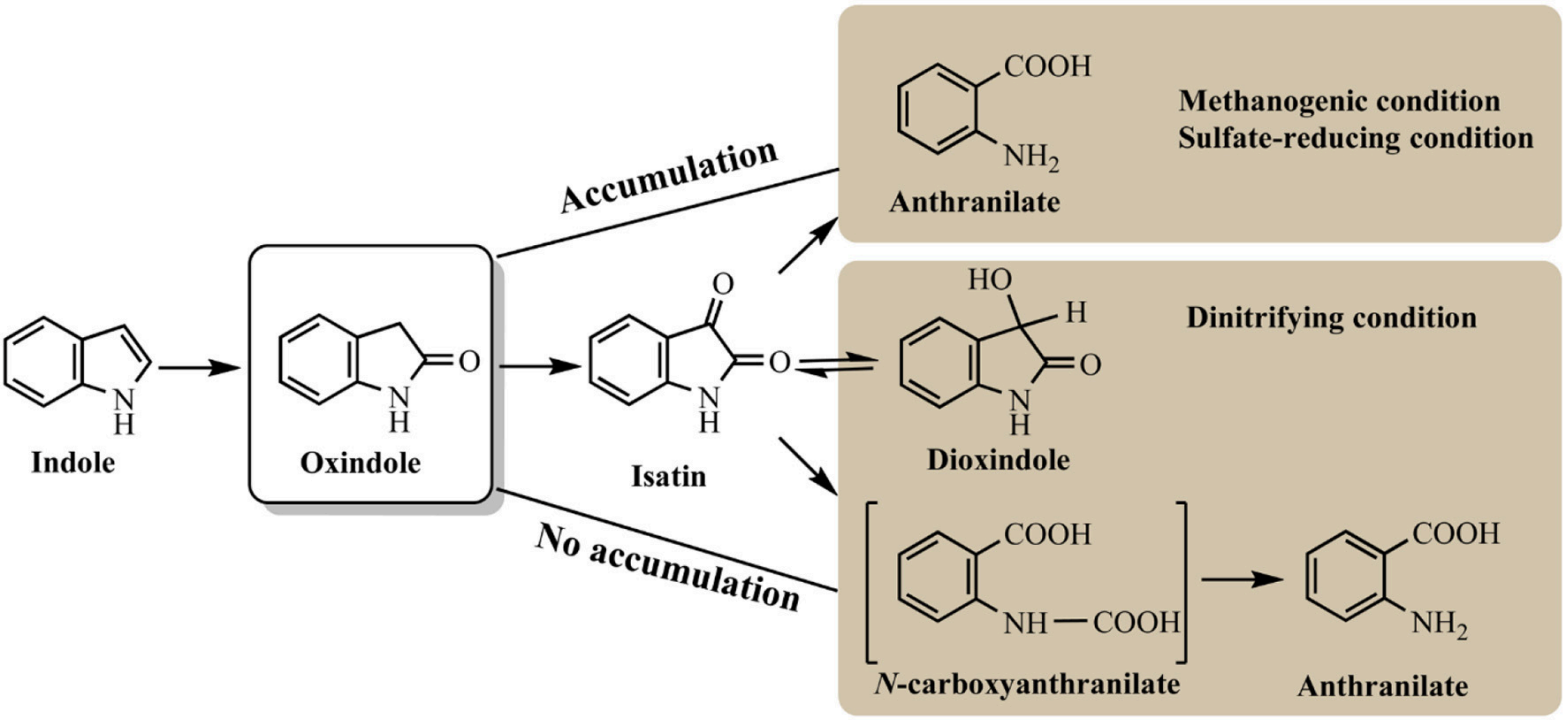
Summary of indole anaerobic pathways under different conditions.

Indole anaerobic degradation studies were concentrated on microbial communities and the initial studies could date back to 1980s. Balba and Evans (1980) investigated the methanogenic fermentation of tryptophan and indole was detected as the key intermediate, which was further converted to anthranilate, salicylate and benzoate. Wang et al. (1984) showed that indole could be degraded under methanogenic conditions and mineralized to methane and carbon dioxide. However, these results were inconclusive due to the sorption phenomena. Berry et al. (1987) confirmed the mineralization of indole to methane with the inoculum of sewage sludge, and oxindole was carefully identified as the degradation intermediate. Further experiment indicated that with the decrease of temperature and concentration of sewage sludge inoculum, oxindole could reach higher concentration and persisted longer under methanogenic conditions (Madsen et al., 1988). Complete mineralization of indole has also been observed under sulfate-reducing conditions, which shared similar pathway with that under methanogenic conditions (Shanker and Bollag, 1990). Gu and Berry (1991) has shown that the substituted groups at indole would affect its degradation rates and pathway. Specially, a methyl group at C1 (1-methylindole) or C2 (2-methylindole) inhibited its hydroxylation and degradation, and 3-methylindole could only be hydroxylated in the 2-position resulting in 3-methyloxindole formation without further mineralization under methanogenic conditions. Indole degradation under denitrifying conditions was carefully conducted by Madsen et al. (1988) with nitrate-reducing sewage sludge. Oxindole appeared only in trace amounts without accumulation possibly due to the exceeded oxindole elimination rate over production rate. High-performance liquid chromatography, thin-layer chromatography, and mass spectrometry analyses identified four key intermediates, i.e., oxindole, isatin, dioxindole and anthranilate, in indole mineralization process in the nitrate-reducing sewage (Figure 5; Madsen and Bollag, 1989).

Pathways for indole aerobic and anaerobic degradation share certain similarities. Hydroxylation of indole is believed to be the essential step in its metabolism. Isatin, dihydroxyindole and anthranilate are the common intermediates detected under both conditions. The obvious difference is that indole is often aerobically oxidized at C3 or C2/C3 position as the first step, which, however, is generally hydroxylated at C2 position leading to 2-oxindole production under anaerobic condition. Under methanogenic and sulfate-reducing conditions, 2-oxindole can be accumulated and easily detected as main intermediate, while it is effectively converted under nitrifying conditions, which may be the reason for the high indole degradation efficiency (Madsen et al., 1988; Shanker and Bollag, 1990). All studies have indicated that benzene ring of indole remained intact and hydroxylation only occurred at pyrrole ring. It can be speculated that functional enzyme for indole anaerobic degradation is distinct from the aerobic ones. The source of oxygen used in the enzyme-mediated oxidation should be from water rather than molecular oxygen, and anaerobic enzyme regioselectively attacked C2 rather than C3 position.

### Indole-degradation strain and microbial community

Indole could be degraded under various anaerobic conditions, while the functional microbes have been sporadically referred. Hong et al. (2010) firstly explored the microbial communities using the 16S rRNA clone library method. It was shown that *Alicycliphilus, Alcaligenes*, and *Thauera* were dominant genera in the denitrifying bioreactor. The major genera were similar with those of aerobic degradation bacterial community, suggesting they were the facultative and functionally important bacteria in indole degradation process. Under sulfate-reducing condition, bacterial community was significantly distinct, and *Clostridia* and *Actinobacteria* were the major classes, which should be resulted from the different electron acceptors of these two systems. Recently, indole degradation in microbial fuel cell (MFC) was achieved with electricity generation (Luo et al., 2010). Up to 88% indole (250 mg/L) could be removed within 3 h, whose rate was much superior to microbial reactions. MFC system provided another avenue for indole-containing wastewater treatment, while the microbial community on the anodes, possibly *Desulfovibrio* related genera, required further investigation.

*Desulfobacterum indolicum* In04 was the first and only isolated bacterium which could degrade indole under strictly anaerobic conditions up to date (Bak and Widdel, 1986). It could completely convert indole to CO_2_ using sulfate as the electron acceptor. The indole degradation intermediates by strain In04 were oxindole, isatin and anthranilate, same with those detected in community systems. Three *P. putida* strains were also isolated from activated sludges that could use indole as sole carbon source under anoxic condition (Li and Young, 2013). Metabolite analyses verified that indole was degraded via the traditional anaerobic pathway, i.e., oxindole, isatin and anthracitic acid. Similar with *Pseudomonas, Thauera* was also reported to be able to degrade indole under both aerobic and anaerobic conditions (Mao et al., 2010). This genus was detected as the main population in the denitrifying bioreactor and acerbic degradation community, further highlighting its versatile indole degradation capacity.

## Indole to indigo: a subject of unfailing interest

### Bacterial strains transforming indole to indigo

Indigo is one of the oldest pigments used in dying, food, pharmaceutical and semiconductor industries, and it is known for the application of producing the popular blue denim. Indigo is traditionally extracted from plants, while the indigo market has been soon taken up by chemical production after Baeyer synthesized the coloring matter in the lab (Huxtable, 2001). However, chemical synthesis will unavoidably use toxic compounds (e.g., aniline) as the substrates and produce industrial wastewater, causing environmental and public health concerns. Microbial production of indigo has been attracting a huge attention in the last few decades due to its environment-benign and mild condition requirements. The formation of indigo was firstly reported in the study of indole degradation process by *P. indoloxidans* (Gray, 1928; Oshima et al., 1965). Two molecules of indole were oxidized to one molecule of indigo and indoxyl was detected as the intermediate in indigo formation reaction. Up to date, there have been amounts of bacterial strains reported with the ability of indigo formation from indole as shown in Table 2.

**Table 2:** Bacterial strains with the ability of producing indigo from indole.

**Wild strains**	**Inducer**	**Indigo production**	**References**
*P. indoloxidans*	Indole	–	Gray, 1928; Oshima et al., 1965
*P. putida* PpG7	Naphthalene	~1.6 mg/L in 30 min by naphthalene induced cells	O'Connor and Hartmans, 1998
*P. putida* RKJ1	Naphthalene	~2.0 mg/L in 30 min by salicylate/naphthalene induced cells	Bhushan et al., 2000
*P. aeruginosa* HOB1	Naphthalene	246 mg/L	Pathak and Madamwar, 2010
*P*. sp. J26	Naphthalene	36.2 mg/L	Mercadal et al., 2010
*P. putida* F1	*p-*Cumate	~1.3 mg/L in 30 min by toluene induced cells	O'Connor and Hartmans, 1998
*P. putida* mt-2	*m*-, *p*-Toluate	~0.1 mg/L in 30 min by toluene induced cells	O'Connor and Hartmans, 1998
*P*. sp. ST-200	Indole	6.2 mg/L	Doukyu et al., 1998
*P. putida* S12	Styrene	~9.8 mg/L in 30 min by styrene induced cells	O'Connor and Hartmans, 1998
*P. putida* CA-3	Styrene	~5.3 mg/L in 30 min by styrene induced cells	O'Connor and Hartmans, 1998
*P. monteilii* QM	Phenol	27.2 mg/L	Ma et al., 2012
*P*. sp. PI1	Phenol	11.8 mg/L	Wang et al., 2015
*A*. sp. ST-550	Phenol	292 mg/L	Doukyu et al., 2002
*A*. sp. PI2	Phenol	17.1 mg/L	Wang et al., 2015
*A*. sp. PP-2	Phenol, etc.	202.9 mg/L	Qu et al., 2010
*Comamonas* sp. MQ	Naphthalene	32.2 mg/l	Qu et al., 2012b
*Nocardia* sp. S3	Styrene	~0.7 mg/L in 30 min by toluene/styrene induced cells	O'Connor and Hartmans, 1998
*Rhodococcus* sp. NCIMB 12038	Naphthalene, etc.	–	Boyd et al., 1997
*Sphingomonas macrogolitabida* TFA	Tetralin	~0.16 mg/L per minute	Moreno-Ruiz et al., 2003

*Pseudomonas*, a typical type of strain for organic compounds biodegradation, was the most reported indigo-producing genus. O'Connor and Hartmans (1998) investigated five *Pseudomonas* strains and found that styrene-induced *P. putida* S12 and CA-3 produced indigo at high rates of 5–10 mg/L in 30 min, while toluene- and naphthalene-induced *P. putida* F1 and PpG7 produced 1.3–1.6 mg/L indigo in 30 min. Afterwards, Bhushan et al. (2000) found that naphthalene-induced *P. putida* PKJ1 could also produce indigo at a relative low rate around ~2.0 mg/L in 30 min. Pathak and Madamwar (2010) isolated an efficient naphthalene degrading strain *P*. sp. HOB1, and it could produce as high as 246 mg/L indigo. We have previously reported that phenol-induced *P*. sp. QM and PI1 could also produce indigo, and other red and purple indigoids were also generated (Ma et al., 2012; Wang et al., 2015).

*Acinetobacter* was another famous indigo-producing genus. Doukyu et al. (2002) obtained *A*. sp. ST-550 from soil sample and it could produce 292 mg/L indigo in the diphenylmethane-water two phase system. Ishikawa et al. (2014) introduced the bacterionanofiber gene *ataA* into strain ST-550 to improve its adhesiveness, and the recombinant strain could be easily immobilized on a polyurethane support. The immobilized strain could tolerant higher concentration of indole and produce more indigo, and this strategy provided a smart way for efficient indigo production and recovery. *A*. sp. strain PP-2 was previously obtained in our lab and could produce indigo induced by different aromatics, such as phenol, naphthalene and xylene (Qu et al., 2010). The indigo formation conditions were optimized and the yield could reach the highest of 202 mg/L when induced by phenol.

### Enzyme resources and indole transformation pathways

Indoxyl is the precursor of indigo, thus indole oxidation to indoxyl is the key step for indigo production. Many oxygenases have been reported to be able to oxidize indole, and these enzymes could be mainly divided into dioxygenase and monooxygenase. The indigo formation enzymes have been summarized in Table 3, and typical oxygenases include naphthalene dioxygenase (NDO), multicomponent phenol hydroxylase (mPH), cytochrome P450 monooxygenase and flavin monooxygenase (FMO). Since indigo pigment produced by *E. coli* strains is visible and can be easily screened from clone libraries, the indigo-screening method has been widely used for isolation of oxygenases both from genome and metagenome libraries (Guan et al., 2007; Van Hellemond et al., 2007; Singh et al., 2010). Although high and pure indigo could be produced by microbial process, the eco-efficiency analysis indicated that bio-indigo production is less efficient than chemical processes (Philp et al., 2013). Nevertheless, this novel and green biological process has attracted numerous attention, and related studies continue today.

**Table 3:** Enzyme resources with the ability of producing indigo from indole.

**Enzyme**	**Sources**	**Characteristics**	**References**
Naphthalene dioxygenase	*P. putida* PpG7	The first reported indigo formation protein	Ensley et al., 1983
	*P*. sp. NCIB9816-4	Production of various indigoids from indole derivatives	Kim et al., 2003
	*P. putida* RKJ1	1.50 nmol/(min·mg dry biomass) indigo	Bhushan et al., 2000
	*Comamonas* sp. MQ	Production of 205 mg/L indigo, and various indigoids from indole derivatives	Zhang et al., 2013
Phenol hydroxylase	*A*. sp. ST-550	52 mg/L indigo	Doukyu et al., 2003
	*P*. sp. KL33	Transformation of indole to 7-hydroxyindole and indole derivatives to various indigoids	Kim et al., 2005
	*P*. sp. KL28	Production of various indigoids from indole derivatives	Kim et al., 2005
	*Arthrobacter* sp. W1	Transformation of indole to indigo, indirubin and 2-(7-oxo-1H-indol-6(7H)-ylidene) indolin-3-one	Qu et al., 2012a
Cytochrome P450 monooxygenase	Human	P450 and mutant P450 2A6 could transform indole and its derivatives to isatin, 6-hydroxyindole, oxindole, indigo and indirubin, and various indigoids	Gillam et al., 2000; Nakamura et al., 2001
	*B. megaterium*	A cofactor regeneration system is constructed in P450 BM-3 to improve indigo production	Lu and Mei, 2007; Hu et al., 2010
Styrene monoxygenase	*P. putida* S12	Indole oxide pathway, and wild strain produces pure indigo	O'Connor et al., 1997
	*P. putida* CA-3	Indole oxide pathway, and wild strain produces pure indigo	O'Connor et al., 1997
	*P. putida* B4	Production of 52.13 mg/L indigo by overexpressing *styAB* in wild strain	Cheng et al., 2016
Flavin monooxygenase	*Methylophaga aminisulfidivorans* MP^T^	As high as 880 mg/L indigo, and 223.6 mg/L indirubin by addition of cysteine	Choi et al., 2003; Han et al., 2013
	*Corynebacterium glutamicum* ATCC13032	Transformation of 2.5 g/L tryptophan to 685 mg/L indigo	America et al., 2015
Acyl-CoA dehydrogenase-like protein	*A. baumannii*	IacA for indole, *K_*m*_* 0.80 mmol/L, *k_*cat*_*0.88 min^−1^	Lin et al., 2012
	*Rhodococcus sp*. T104	IpoA loses indigo-producing ability by mutation at non-active site	Kwon et al., 2008
	*Ralstonia eutropha* HF39	Indigo inclusions have a diffuse structure in the cells	Drewlo et al., 2001
	*P. alcaligenes* PA-10	IdoA is the key protein constitutively expressed for fluoranthene metabolism	Alemayehu et al., 2004
Indole oxygenase	*A. baumannii* ATCC19606	IifC for indole, *K_*m*_* 0.20 mmol/L, *k_*cat*_* 0.38 min^−1^	Lin et al., 2015
	*A*. sp. O153	IifC for indole, *K_*m*_* 0.25 mmol/L, *k_*cat*_* 0.11 min^−1^	Sadauskas et al., 2017
	*C*. sp. SHE	IndA for indole, *K_*m*_* 0.36 mmol/L, *k_*cat*_* 1.53 min^−1^	Qu et al., 2017
Metagenomic protein	Forest soil	64 mg/L indirubin and 34 mg/L indigo	Lim et al., 2005
	Loam soil	SmoA is a novel type of styrene monooxygenase	Van Hellemond et al., 2007
	Midguts of gypsy moth larvae	MoxY produces isatin, indigo and indirubin	Guan et al., 2007
	Activated sludge	B1 and B2 can produce indigo and indirubin	Singh et al., 2010
Xylene monooxygenase	*P. putida* mt-2	First reported indigo formation monooxygenase	Mermod et al., 1986
Toluene dioxygenase	*P. putida* F1	Transformation of indole derivatives to various indigoids	Kim et al., 2003
Toluene 2-monoxygenase	*Burkholderia cepacia* G4	Transformation of indole derivatives to various colorful products	Rui et al., 2005
Toluene 4-mooxygenase	*P. mendocina* KR1	Transformation of indole derivatives to various indigoids	McClay et al., 2005
2-hydroxybiphenyl 3-monooxygenase	*P. azelaica* HBP1	Mutant HbpA_ind_ transforms indole derivatives to various indigoids	Meyer et al., 2002
Biphenyl dioxygenase	*Dyella ginsengisoli* LA-4	44 mg/L indigo	Qu et al., 2013

**Figure 6:**
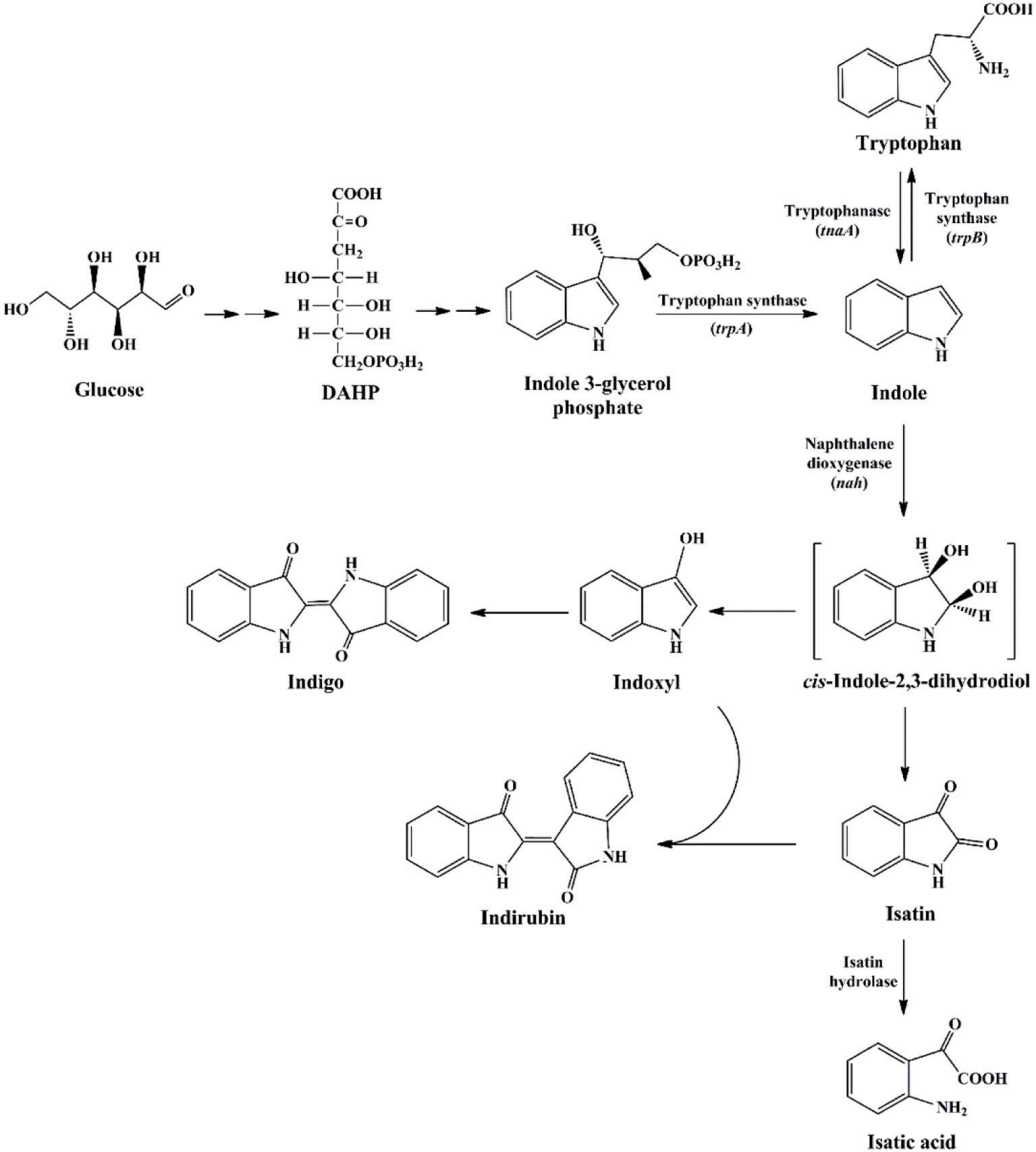
Indigoids biosynthesis pathway by naphthalene dioxygenase.

The first as well as the most reported oxygenase in indigo production field is NDO. Ensley et al. (1983) cloned an NDO gene from *P. putida* PpG7 and expressed it in *E. coli*. The recombinant strain was able to produce indigo, an unnatural end product of *E. coli*, in nutrient medium. Analysis indicated that *E. coli* could convert tryptophan to indole by tryptophanase, which was further oxidized to indoxyl by NDO, leading to the production of indigo (Figure 6). This study initiated the indigo bioproduction history. Ten years later, Amgen industry successfully constructed the first metabolic pathway catalyzing indigo biosynthesis from glucose in *E. coli* (Murdock et al., 1993; Bialy, 1997). The activity and half-life time of NDO was significantly improved by promoter and ferredoxin gene component modification, and then high level of indole was produced from glucose in *E. coli* by inactivation of the β subunit of tryptophan synthetase. However, the indigo yield, 135 mg/L in lab experiment, was not satisfactory for commercial application. Almost another 10 years later, scientists from Genencor International reconstructed the fermentation process based on metabolic engineering to improve the production of bio-indigo (Berry et al., 2002). A high-level indigo producing strain was developed designated as FM5/pBMW, pTacFd911. The production of indigo was further enhanced by improving 3-deoxy-D-arabino-heptulosonate 7-phosphate (DAHP) formation via several strategies, reaching 23 g/L in 42 h. Indirubin was a common byproduct usually co-produced in indigo production process. It was proposed that indoxyl and isatin/2-hydroxyindole were the substrate for indirubin formation. The serious and common by-product problem was resolved by incorporation of an isatin hydrolase, which could open the pyrrole ring of isatin and block indirubin production. In addition to indole, it has been proved that NDO could convert various indole derivatives, including methylindoles, methoxyindoles, nitroindoles, chloroindioles, and bromoindoles, to different indigoid pigments (Kim et al., 2003; Zhang et al., 2013).

**Figure 7:**
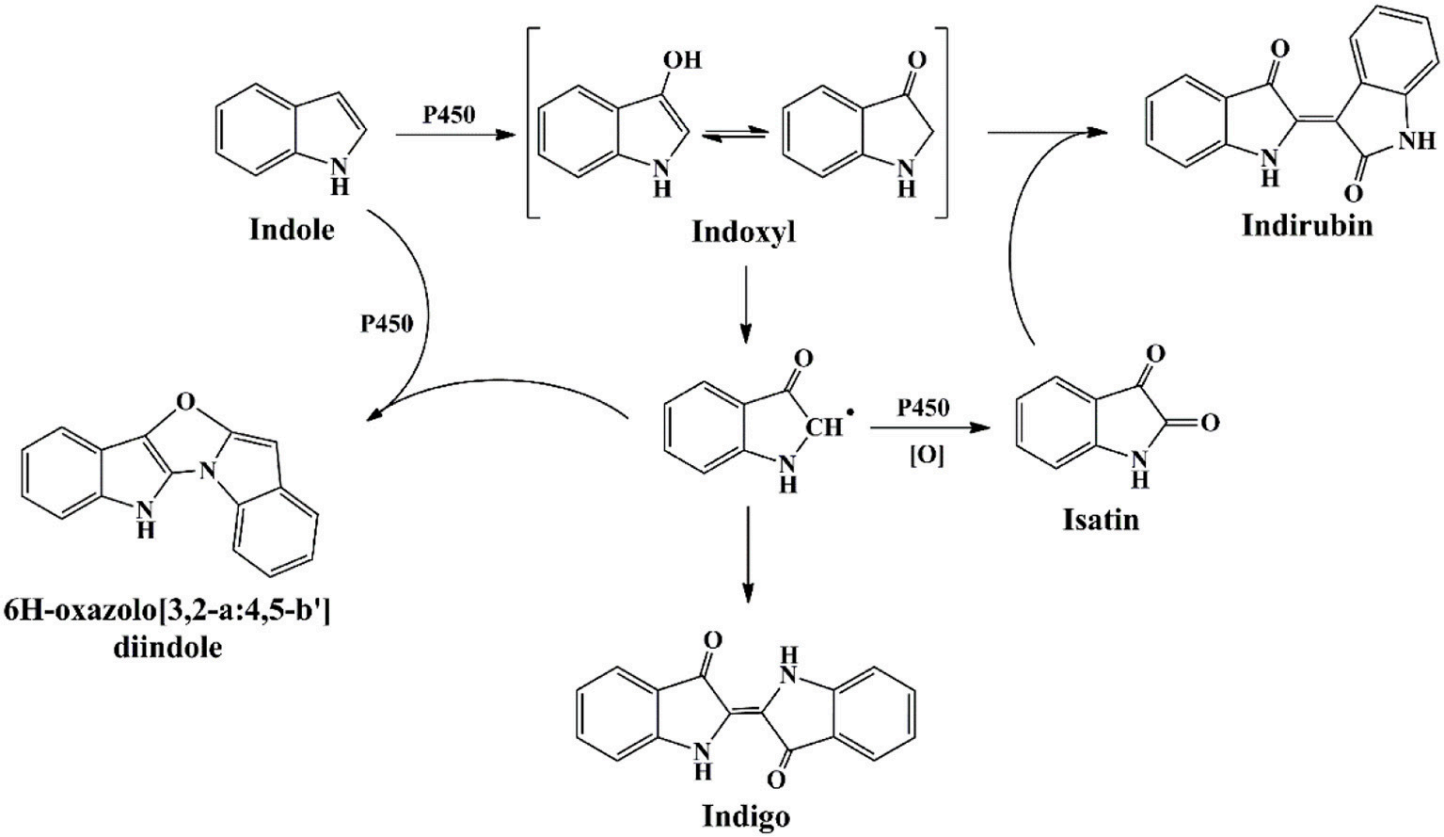
Indigoids biosynthesis pathway by cytochrome P450 monooxygenases.

Cytochrome P450 enzymes have been found in all kingdoms of life, including bacteria, fungi, plants, animals and human beings. They are among the most versatile proteins with the capability of catalyzing C-hydroxylation, heteroatom oxygenation, heteroatom release, and epoxide formation, and have been widely applied in chiral compounds production, drug development and biodegradation fields (Urlacher and Girhard, 2012). Guengerich group firstly reported and undertook massive work in the indigo formation by cytochrome P450 enzymes (Gillam et al., 1999). P450 2A6 was active in indole biotransformation and produced indigo, indirubin and a new product 6H-oxazolo[3,2-a:4,5-b′]diindole (Figure 7) (Gillam et al., 2000). Random mutagenesis of P450 2A6 resulted in some mutants with higher indigo production capacities, and the mutants could convert substituted indoles to different indigoids. The 5-methylindole transformation product, i.e., 5-methylindirubin, 5′-methylindirubin and 5,5′-dimethoxyindirubin, showed highest inhibition activity toward human cyclin-dependent kinases (CDK)-1 and - 5 and glycogen synthase kinase-3 (GSK3), facilitating the research in drug development (Guengerich et al., 2004). Lu and Mei (2007) co-expressed the P450 BM3 and glucose dehydrogenase and improved the indigo yield. Random and saturation mutagenesis produced several mutants with higher indole oxidation performance, and the mutant D168W could produce indirubin as the main product (90%) rather than indigo by the wild P450 BM3 enzyme, providing novel information for indirubin bio-production (Hu et al., 2010).

**Figure 8:**
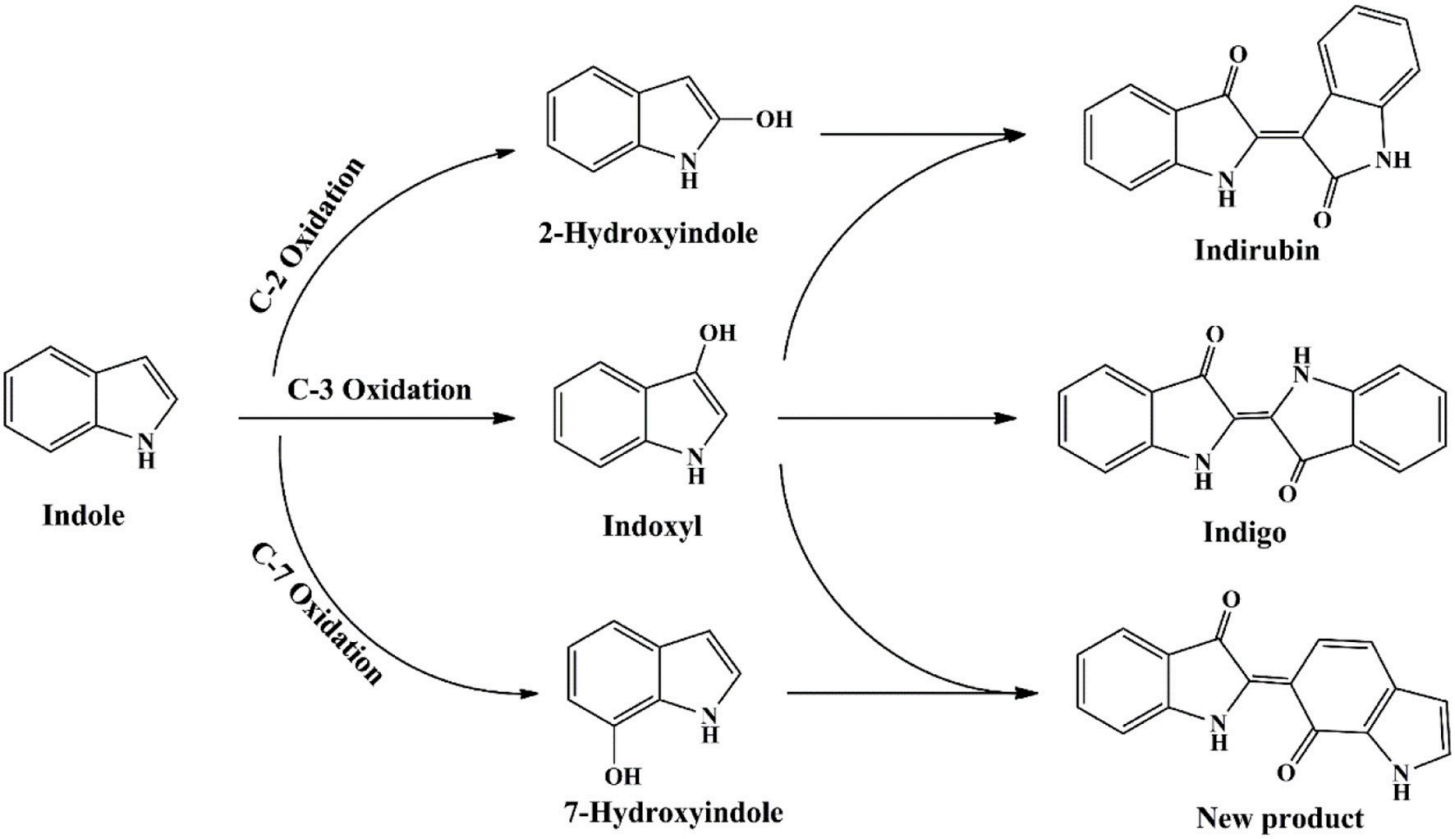
Indigoids biosynthesis pathway by phenol hydroxylase.

The functional enzyme mPH was firstly identified in *P*. sp. CF600 in phenol and (di)methylphenol degradation process, which was encoded by six genes *dmpKLMNOP* (Nordlund et al., 1990). Doukyu et al. (2003) has cloned the mPH gene from *A*. sp. ST-550 and co-expressed with NAD-dependent formate dehydrogenase gene, and the host cell could produce 52 mg/L indigo. Kim et al. (2005) expressed two different mPHs from *P*. sp. KL33 and *P*. sp. KL28. The mPH_KL28_ mainly produced indigoid pigments from indole and could catalyze the formation of various dyestuffs from a wide range of indoles. However, the mPH_KL33_ converted indole to 7-hydroindole. Our previous study indicated that the mPH from *Arthrobacter* sp. W1 could transform indole to three pigments, i.e., indigo (blue), indirubin (red) and 2-(7-oxo-1H-indol-6(7H)-ylidene) indolin-3-one (purple) (Qu et al., 2012a). It was reported that many other phenol-degrading bacteria, including *P*. sp. CF600, *P*. sp. KL33, and *P*. sp. QM, converted indole to deep purple or red-purple pigments (Kim et al., 2005; Ma et al., 2012). 7-Hydroindole was identified as the intermediate in these strains, suggesting the purple products might contained 2-(7-oxo-1H-indol-6(7H)-ylidene) indolin-3-one, which was derived from indoxyl and 7-hydroindole. The proposed indole transformation pathway by mPH should cover three pathways, i.e., C2, C3, and C7, distinct from other oxygenases (Figure 8) (Qu et al., 2012a).

**Figure 9:**
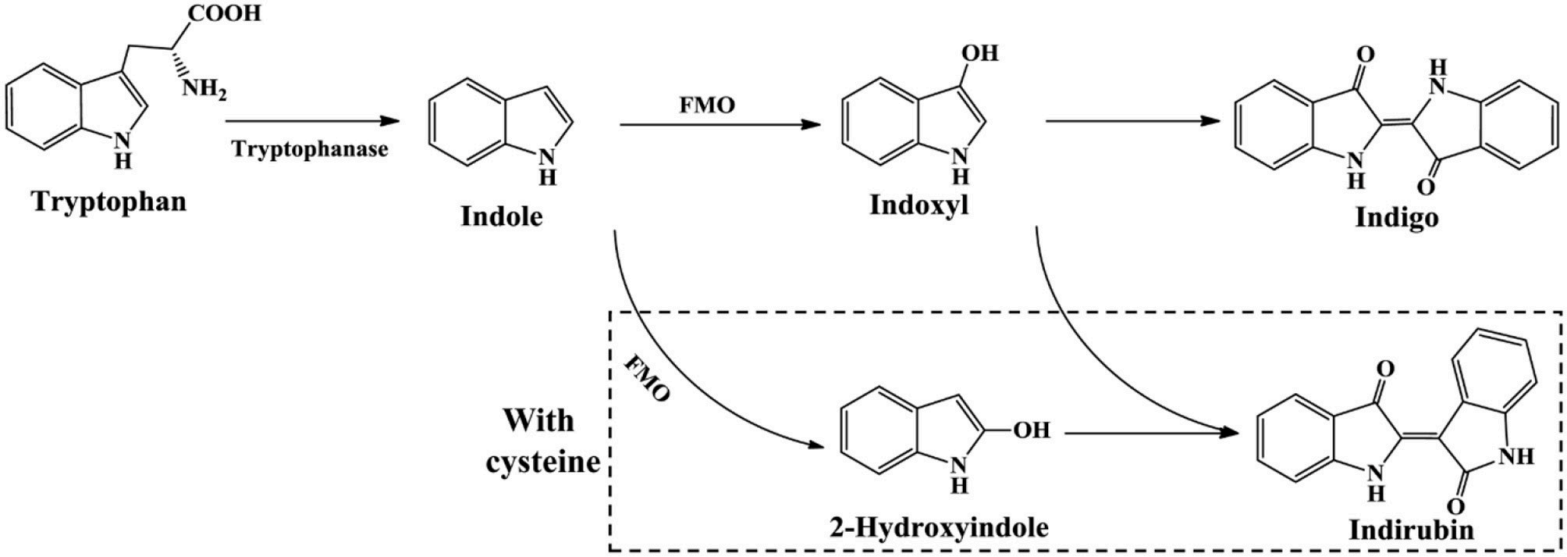
Indigoids biosynthesis pathway by flavin-containing monooxygenase.

The newly reported indigo-producing enzyme FMO was identified from strain *Methylophaga aminisulfidivorans* MP^T^ (Choi et al., 2003). The recombinant *E. coli* strain expressing *fmo* gene could originally produce 160 mg/L indigo in tryptophan medium. Subsequently, the upstream sequence of *fmo* and the culture medium composition were optimized by surface methodology method. The maximum yield of bio-indigo reached as high as 920 mg/L in the 5-L and 3000-L fermentation system under optimal conditions, the highest yield in batch fermentation system up to date (Han et al., 2008). In addition, Han et al. (2013) found that addition of cysteine could lead to the 2-hydroxyindole formation from indole, which significantly enhanced indirubin production (Figure 9). This finding provided a new and simple approach for the production of valuable indirubin.

### Smart strategy for indigo dying process

**Figure 10:**
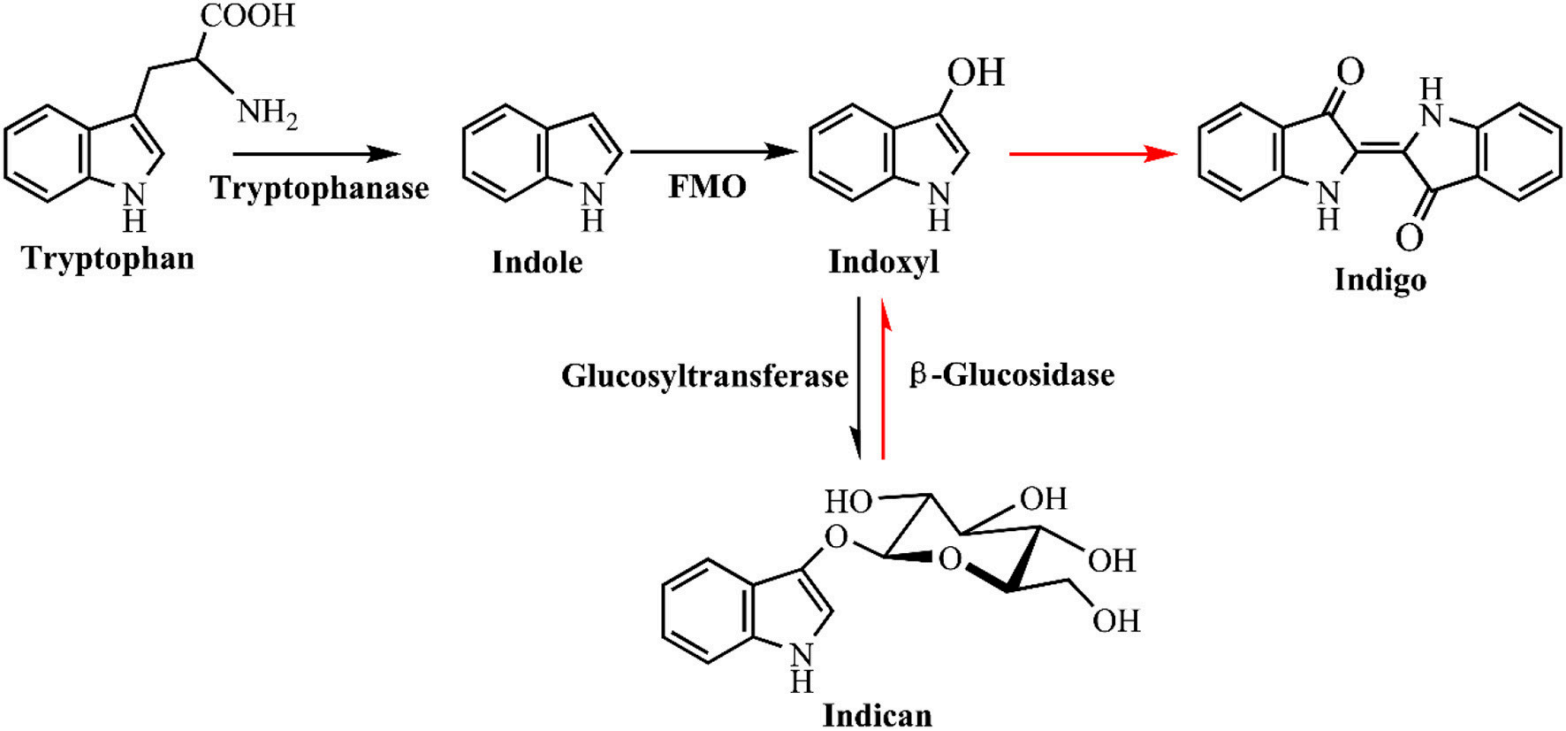
A novel glucosyl protecting group based indigo production and dying strategy.

Microbial production of indole has solved the problem of hazardous and toxic reagents used in indigo chemical synthesis, while the indigo dying process is still environmentally unfriendly. Indigo is insoluble in water and excess reducing agent, such as sodium dithionite, must be added to covert indigo to leuco form before dying. Hsu et al. (2018) has developed a two-step sustainable biochemical route to solve the indigo reducing problem, as shown in Figure 10. They firstly obtained a glucosyltransferase from Japanese indigo plant *Polygonum tinctorium*. Expression of the glucosyltransferase gene and *fmo* gene in *E. coli* could successfully lead to indicant production rather than the unstable indoxyl. The glucosyl group in indicant could be enzymatically hydrolyzed by a β-glucosidase to produce indoxyl and leucoindigo whenever needed, thus the dying process could avoid the reducing step. Compared with indoxyl, indican is stable, water soluble, easily concentrated and suitable for long storage. Although some challenges still exist for practical dying process, this indoxyl glucosylation method is historic and remarkably promotes bio-indigo application.

## Future perspectives

**Figure 11:**
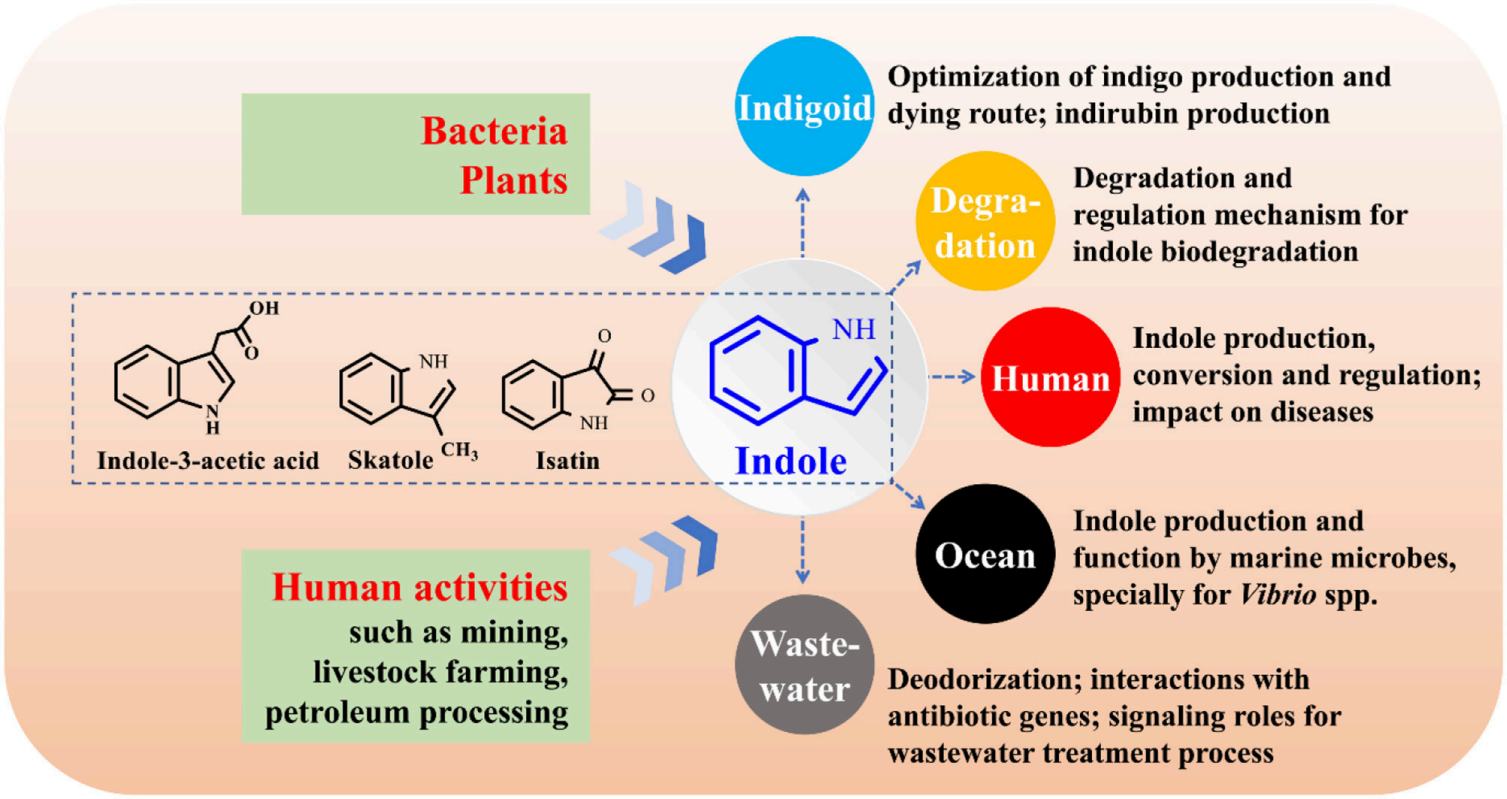
Research perspectives on indole-microbe interactions.

The function study of indole as an interspecies and interkingdom signaling molecule has been a hot topic, especially its impact on bacterial antibiotic resistance, virulence, and persister formation (Lee and Lee, 2010; Lee et al., 2015a). Bacteria, plants and anthropic activities can produce considerable amounts of indole and release it into environment. Bacteria will inevitably encounter indole, while the interactions between indole and its surrounding environments largely remain uncovered. The following aspects on indole-microbe interactions beyond signaling research are worthy of further investigation (Figure 11).

1. Industrial application of indigoids bio-production. More efficient enzymes and pathways for indole biotransformation are to be constructed, the downstream procession of bio-indigo requires attention, and the challenge of indirubin biosynthesis needs in-depth investigation.
2. Molecular mechanisms for indole degradation. The enzymes besides indole oxygenase for indole oxidation in some bacteria remain unknow. Formation and transformation of isatin and anthranilate in indole degradation may be strain-dependent and remain controversial, and the regulation of indole metabolic genes are rarely referred. Furthermore, the indole degradation mechanism under anaerobic conditions has still been a black box.
3. Impacts of indole on human health. Indole, derived from tryptophan in food via microbial transformation, is rich in animal and human gut (Lee et al., 2015a). However, the substrate tryptophan can be also converted to indole-3-acetic acid and skatole (Deslandes et al., 2001). The functional strains and regulation of the transformation process in intestinal tracts are still unknown. Indole may impact several diseases such as inflammatory bowel diseases and diabetes (Bansal et al., 2010; Chimerel et al., 2014). However, solid evidences of indole roles should be further provided and the functional mechanism urgently needs to be solved. Indole has been proven to be able to extend healthspan (remain healthy and free of age-related infirmities) in animals by interacting with the aryl hydrocarbon receptor (Sonowal et al., 2017), highlighting the potential of indoles as new therapeutics to improve life quality and reduce frailty.
4. Functions of indole on marine organisms. Marine bacteria can produce indole, while the researches of indole production by marine bacteria are numbered (Takao et al., 1994; Lee and Lee, 2010). Practically in marine aquaculture industries, tryptophan is abundant in aquaculture feed, and the indole-producing *Vibrio* strains are the most common pathogenic marine bacteria. Thus, it can be inferred that indole is spread in aquaculture systems. Indole has been found to control the biofilm and virulence of *Vibrio* strains, and it may also regulate the antibiotic resistance and virulence of other indole-producing and non-indole-producing marine strains (Mueller et al., 2009). Therefore, indole may contribute to, negatively or positively, the aquaculture diseases, antibiotic resistance gene (ARG) expression and propagation.
5. Impacts of indole on the waste treatment process. Treatment of indole-containing wastewater is always accompanied by indigoids formation, thus isolation of non-indigo production indole-degrading bacteria or genetically modification of bacterial metabolic pathways are feasible solutions (Lee et al., 2015a). Indole as a signaling molecule can coordinate cell-cell communication in microbial communities. Quorum sensing (QS), the most well studied cell-cell communication mechanism, has been found to play crucial roles in wastewater treatment process via control of microbial adhesion, colonization, and biofilm formation, while bacterial QS of indole in these systems has yet been concerned (Yeon et al., 2008; Feng et al., 2013). Indole and skatole are the major contributor to boar taint and the malodor emission from livestock waste and human feces (Mackie et al., 1998; Hales et al., 2012), and development of microbial agents and enzymes will have a broad application prospect in livestock industry. More importantly, livestock wastes are the major sources of ARG which may pose potential worldwide human health risks (Zhu et al., 2013). Indoles in livestock wastes can affect the antibiotic resistance and virulence of several pathogens, influencing the ARG richness and antibiotic usage. Indoles-containing wastewater and manure will enter river, soil and farmland, further interacting with ARG, and the impacts deserve to be investigated.

## Author contributions

QM wrote the paper, and YQ and XZ revised the paper. All authors approved it for publication.

### Conflict of interest statement

The authors declare that the research was conducted in the absence of any commercial or financial relationships that could be construed as a potential conflict of interest.
